# Unravelling roles of error-prone DNA polymerases in shaping cancer genomes

**DOI:** 10.1038/s41388-021-02032-9

**Published:** 2021-10-18

**Authors:** Cyrus Vaziri, Igor B. Rogozin, Qisheng Gu, Di Wu, Tovah A. Day

**Affiliations:** 1 Department of Pathology and Laboratory Medicine, University of North Carolina at Chapel Hill, 614 Brinkhous-Bullitt Building, Chapel Hill, NC 27599 USA; 2 National Center for Biotechnology Information, National Library of Medicine, National Institutes of Health, Bethesda, MD 20894 USA; 3 Department of Biostatistics, University of North Carolina at Chapel Hill, 135 Dauer Drive, 3101 McGavran-Greenberg Hall, Chapel Hill, NC 27599 USA; 4 Department of Biology, Northeastern University, Boston, MA 02115 USA

**Keywords:** Sequencing, Cancer genetics

## Abstract

Mutagenesis is a key hallmark and enabling characteristic of cancer cells, yet the diverse underlying mutagenic mechanisms that shape cancer genomes are not understood. This review will consider the emerging challenge of determining how DNA damage response pathways—both tolerance and repair—act upon specific forms of DNA damage to generate mutations characteristic of tumors. DNA polymerases are typically the ultimate mutagenic effectors of DNA repair pathways. Therefore, understanding the contributions of DNA polymerases is critical to develop a more comprehensive picture of mutagenic mechanisms in tumors. Selection of an appropriate DNA polymerase—whether error-free or error-prone—for a particular DNA template is critical to the maintenance of genome stability. We review different modes of DNA polymerase dysregulation including mutation, polymorphism, and over-expression of the polymerases themselves or their associated activators. Based upon recent findings connecting DNA polymerases with specific mechanisms of mutagenesis, we propose that compensation for DNA repair defects by error-prone polymerases may be a general paradigm molding the mutational landscape of cancer cells. Notably, we demonstrate that correlation of error-prone polymerase expression with mutation burden in a subset of patient tumors from The Cancer Genome Atlas can identify mechanistic hypotheses for further testing. We contrast experimental approaches from broad, genome-wide strategies to approaches with a narrower focus on a few hundred base pairs of DNA. In addition, we consider recent developments in computational annotation of patient tumor data to identify patterns of mutagenesis. Finally, we discuss the innovations and future experiments that will develop a more comprehensive portrait of mutagenic mechanisms in human tumors.

## Introduction

Mutagenesis is a hallmark and enabling characteristic of cancer. Accumulation of mutations permits neoplastic cells to adapt to their environments, evolve, resist therapies, and potentially develop neoantigens which can promote disease and/or therapeutic resistance. Further, mutagenesis can result in therapy-induced secondary neoplasia. Therefore it is critical to understand the error-prone DNA repair and replication mechanisms that generate mutations and to determine the extent to which mutational scars of cancer cells have biomarker, predictive, or prognostic value to guide therapeutic decisions. In this review, we define ‘error-prone DNA polymerases’ as those that exhibit reduced fidelity when copying an undamaged, B-form DNA template including the translesion synthesis (TLS) polymerases, and Pols θ, β, λ, μ, ν, and Primpol. The TLS polymerases (Pol η, Pol ι, and Pol κ, REV1, and Pol ζ), important for replicating past exogenous DNA lesions and endogenous DNA obstacles (e.g., fragile sites) are perhaps the most well-studied subset of error-prone DNA polymerases.

Recent years have seen considerable progress in the enzymology of error-prone DNA polymerases and regulation of their corresponding DNA repair pathways. In parallel, tremendous advances have been made in methodology—both experimental and computational—for identifying and cataloguing patterns of mutations in neoplastic cells. However, the underlying molecular etiology of mutational patterns in human tumors remains incompletely understood. Despite promising advances, we remain at the early stages of experimental validation of observed mutation patterns [1]; the mechanism of almost one-third of all cancer mutational signatures is not yet known [2] while others exhibit complexity that requires further dissection.

An emerging challenge is to determine how DNA repair pathways act upon specific forms of DNA damage and endogenous DNA obstacles to generate cancer-relevant mutations. This problem requires experimental models to recapitulate the mutations found in tumors. As DNA polymerases (Table 1), especially error-prone ones, are the ultimate mutagenic effectors of DNA damage tolerance and repair pathways, investigating their contribution to patterns of mutagenesis is a critical first step in this endeavor. This review summarizes the range of experimental approaches that have investigated and connected DNA polymerases with specific mechanisms of mutagenesis.

**Table 1:** Mammalian DNA polymerases.

Polymerase	Official symbol	GeneID	Family	Canonical pathway assignment
Pol ε	POLE	5426	B	Replication (leading strand)
Pol δ (POLD1, POLD2, POLD3, POLD4)	POLD1, POLD2, POLD3, POLD4	5424, 5425, 10714, 57804	B	Replication (lagging strand)
Pol α	POLA1	5422	B	Replication (RNA primer during DNA replication)
Pol ζ (REV3L, REV7 (aka MAD2L2), POLD2, POLD3)	REV3L, MAD2L2	5980, 10459	B	TLS extension
Pol γ	POLG	5428	A	Replication (mitochondrial)
Pol θ	POLQ	10721	A	TMEJ
Pol ν	POLN	353497	A	End processing?
Pol β	POLB	5423	X	BER
Pol λ	POLL	27343	X	BER
Pol μ	POLM	27434	X	NHEJ
TdT	DNTT	1791	X	NHEJ
Pol η	POLH	5429	Y	TLS
Pol ι	POLI	11201	Y	TLS
Pol κ	POLK	51426	Y	TLS
REV1	REV1	51455	Y	TLS
Telomerase	TERT	7015	RT	End replication (telomere)
PrimPol	PRIMPOL	201973	PrimPol	?

*TLS* trans-lesion synthesis, *TMEJ* theta-mediated end joining, *NER* nucleotide excision repair, *BER* base excision repair, *NHEJ* non-homologous end joining.

## Mechanisms of polymerase dysregulation

**Fig. 1:**
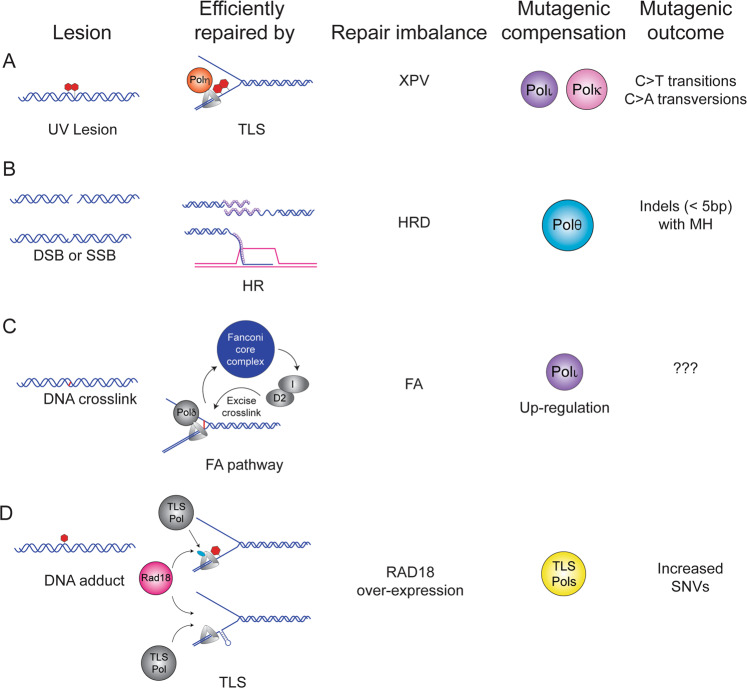
Mutagenic polymerase compensation. **A** UV-induced pyrimidine dimers are efficiently and correctly bypassed by Translesion Synthesis (TLS) using Pol η. In the absence of Pol η, Pol κ, and Pol ι compensate to bypass the lesions leading to a characteristic pattern of C to T transitions and C to A transversions (COSMIC signature SBS7a-c) [3, 4, 145]. **B** Double-strand breaks (DSBs) or single-strand breaks (SSBs) that are processed to a DSB are efficiently repaired by homologous recombination (HR). Germline or somatic mutation or down-regulation of HR factors including BRCA1, BRCA2, BARD1, or Rad51 underlies HR-deficiency (HRD) found in ovarian and pancreatic tumors [146]. In HR-compromised tumors, compensatory DSB repair by Pol θ generates small insertions and deletions with a characteristic microhomology (MHD) signature categorized as the small Insertions and Deletions 6 and 8 (ID6) signature [19, 103]. **C** DNA crosslinks are repaired efficiently by the Fanconi anemia (FA) pathway and germline mutations in one of the 7 components of the Fanconi core complex leads to FA, a tumor-prone disorder. A recent study found that the TLS Pol ι is upregulated in FA cells [50] and it will be interesting to determine whether this contributes to patterns of mutations in FA cells. **D** Activation of the TLS pathway can occur when the replication fork encounters a bulky DNA adduct or an endogenous obstacle to replication such as structured DNA (e.g., a fragile site). The E3 ligase Rad18 mono-ubiquitinates PCNA to recruit the appropriate TLS polymerase to bypass the lesion efficiently. Analysis of TCGA datasets reveals a positive association between *RAD18* expression and overall SNV burden in several tumors including lung adenocarcinoma, lung squamous cell carcinoma, and kidney renal clear cell carcinoma (KIRC) (Table 2) [58] suggesting that dysregulation of TLS leads to mutagenesis.

Mutations in cancer genomes can be a consequence of pathologically-altered DNA repair pathway choice favoring the use of error-prone DNA polymerases in lieu of the default error-free enzymes. This paradigm is well-illustrated by sunlight-sensitive and skin cancer-prone *xeroderma pigmentosum* Variant (XP-V) patients in which the Y-family TLS polymerase Pol η is functionally inactivated. Pol η is specialized to replicate UV-damaged DNA templates that harbor a cyclobutane pyrimidine dimer (CPD) in a relatively error-free manner. Therefore, in XP-V cells lacking Pol η, compensatory error-prone bypass of CPD lesions by Pol ι and Pol κ acting on their non-cognate lesions can lead to mutagenesis while failing to fully compensate Pol η TLS activity [3–5] **(**Fig. 1A).

Historically, Y-family TLS DNA polymerases were thought to be deployed solely for replicative bypass of bulky DNA adducts. However, it is increasingly apparent that they play additional roles in DNA synthesis under a variety of stressful conditions. For example, Pol κ is important for DNA synthesis in low-nucleotide environments resulting from Hydroxyurea (HU) treatment [6]. Nucleotide-deficiency has been proposed as a mechanism of oncogene-induced DNA replication stress [7, 8] and may therefore contribute to the TLS pathway activation observed in oncogene-expressing cells [9–11]. Several TLS polymerases including Pol η, Pol κ, and REV1 are important for sustaining DNA replication [9, 10] and preventing accumulation of ssDNA gaps in oncogene-expressing cells [11]. Additionally, TLS polymerases are important for the replication of DNA fragile sites [12–15]. DNA replication stress, nucleotide deficiency, and fragile site breakage are all hallmarks of neoplastic cells. Therefore, TLS polymerases enable tolerance of intrinsic stresses that arise during tumorigenesis. Owing to their high error-propensity (even when replicating undamaged DNA templates), TLS polymerases are likely key contributors to oncogene-induced mutagenic signatures. In support of this model, in vivo evidence for Pol κ activity was observed at poly dT sites of replication fork stalling and DSB collapse in primary murine cells [16]. Remarkably, the in vivo observation of dinucleotide mutations of CC:GG interrupting a poly dT sequence reflects the in vitro mutagenesis pattern observed for Pol κ in replication of microsatellites [17]. Experimental approaches to define the contributions of Y-family TLS polymerases to mutational spectra of neoplastic cells are discussed later in this review.

By analogy to mutagenic polymerase compensation in XP-V cells, the mutational signatures of homologous recombination deficient (HRD) tumors also arise from imbalance between error-prone and error-free DNA polymerase activities. In mammalian cells, multiple polymerases have been suggested to play a role in HR including Pols θ, δ, ε, ν, ζ, η, κ, and Rev1 [18]. In HRD tumors, compensatory DSB repair by Pol θ generates small insertions and deletions with a characteristic microhomology (MHD) signature categorized as the small Insertions and Deletions 6 and 8 (ID6) signature [19] (Fig. 1B). In addition to the these insertions and deletions (indels), genomes from HRD tumors show increased contribution from COSMIC (Catalogue of Somatic Mutations in Cancer) Single Base Substitution signature 3 (SBS3) [19]. The identity of the DNA polymerase that generates this clinically significant mutational pattern remains unknown although recent data from yeast suggest that Pol ζ may play a role [20].

Arguably then, mutational portraits of cancer cells can be determined by the relative balance between the entire repertoire of available error-free and error-prone DNA repair and tolerance pathways and their effector DNA polymerases. DNA repair imbalance and mutagenesis might result from functional inactivation of error-free pathways (e.g., loss of Pol η in XP-V patients or HRD in breast cancer) and compensation by error-prone mechanisms. Alternatively, an imbalance could potentially result from pathological over-activity of pathways employing error-prone DNA polymerases. There are at least three general ways in which DNA polymerase activities might be pathologically stimulated, leading to error-propensity and mutations:

### (i) Mutations or polymorphisms in DNA polymerases

It is firmly established that replicative DNA polymerases become error-prone through loss of exonuclease domains (residing in amino acids 268–471 of Pol ε and 304–533 of POLD1) which compromise proof-reading activity and predispose to mutagenic activity and cancer [21]. Indeed, mutations in polymerase genes have been linked to both hereditary and sporadic tumors. Evidence from patients with germline variations in polymerase genes indicates that aberrant polymerase activity can contribute to mutagenesis and tumorigenesis. For example, specific germline variants in Pol ε and Pol δ, the leading and lagging strand replicative polymerases, increase susceptibility to early onset colorectal cancer and endometrial cancer [21–26] with many of the variants mapping to the exonuclease domain that confers proof-reading ability. Additional work identified increased risk of a broader spectrum of tumors including brain, breast, skin, pancreatic, and ovarian tumors [27–30]. Notably, two studies found germline mutations in Pol ε in pediatric tumors [31, 32] one of which exhibited an ultra hyper-mutated phenotype [32]. Unsurprisingly, somatic mutations in Pol ε have also been identified in a subset of endometrial and colorectal tumors [33–35] with additional case reports in other types of tumors as well [36, 37]. One study reported that a significant portion of endometrial and colorectal malignancies were correlated with low expression of Pol ε or Pol δ raising the interesting possibility that inappropriately low dosage of replicative polymerases could be mutagenic [25]. Germline or sporadic mutation of replicative polymerases to lower fidelity enzymes can be seen as conversion of high-fidelity polymerases to error-prone polymerases that cells have no choice but to use for replication of the genome.

There is also recent evidence that polymorphisms in Y DNA polymerase families are associated with increased mutagenic activity. For example, some cancer-associated REV1 variants have modest alterations in biochemical activities (including k_cat_/K_m_ for dCTP insertion and DNA-binding affinity) and could contribute to mutability and carcinogenesis [38]. REV1 and Pol κ variants are both reportedly linked with lung cancer susceptibility and survival [39]. Pol κ variants are associated with risk of breast cancer [40] and a Pol ι variant is linked with adenocarcinoma and squamous cell carcinoma [41]. While a limited number of epidemiological studies suggest polymorphisms in Y-family TLS DNA polymerases are associated with cancer risk, further work is needed to determine the extent to which cancer-associated variants impact the mutational landscape.

### (ii) DNA polymerase over-expression

Aberrant high expression of error-prone DNA polymerases has been noted in tumors and may represent a potential mechanism of mutagenesis in cancer cells. However, over-abundance of any error-prone DNA polymerase alone may be insufficient to lead to its engagement with the replisome. Whether an aberrantly overexpressed polymerase is mutagenic may depend on the mechanism by which that enzyme is normally recruited to template DNA. For example, if recruitment of error-prone and error-free DNA polymerases to replicating DNA is stochastic and passive, based solely on polymerase availability and competition for PCNA-binding, then increased expression of an error-prone enzyme may favor its preferential recruitment, favoring mutagenesis. On the other hand, if there is an active DNA polymerase selection and recruitment process, then error-prone DNA polymerase abundance may not be consequential when the recruitment mechanism is rate limiting.

It is interesting to consider the consequences of imbalance between Y-family TLS polymerases as they use both PIP boxes and ubiquitin-binding motifs to associate with mono-ubiquitinated PCNA. Because Y-family TLS polymerases use a shared mechanism to associate with replisomes, it is likely that increased expression of any individual Y-family DNA polymerase would provide a competitive advantage for engaging with the replisome. Although there has been no systematic study of Y family polymerases in cancer, aberrant high expression of specific Y-family TLS DNA polymerases has been noted in certain tumors. For example, Pol ι, a highly mutagenic enzyme with error rates of up to 10^4^ on undamaged DNA templates [42], is reportedly overexpressed in a range of tumor types [43]. Another Y-family DNA polymerase, Pol κ is overexpressed in lung cancer [44] and both Pol κ and Pol ι (but not Pol η) are overexpressed in human gliomas [45].

Whether altered expression of Y-family polymerases necessarily contributes to the mutational portraits of those cancers has not been demonstrated. However, Pol ι over-expression in breast cancer cells is associated with mutagenesis [46] and correlates with clinical tumor grade in bladder cancer [47]. Pol κ overexpression in cultured cells induces spontaneous mutagenesis [44] while transgenic overexpression of Rev1 in mice accelerates the formation of N-methyl-N-nitrosurea (MNU)-induced intestinal adenomas [48], although it does not affect spontaneous tumorigenesis. Therefore aberrant expression of Pol ι, Pol κ, REV1, and possibly other TLS polymerases may promote mutagenesis in tumor cells.

Aberrant expression of other DNA polymerase families may also be relevant to the mutational profiles of cancer cells. Ceccaldi et al. reported an inverse correlation between Pol θ expression and HR activity in epithelial ovarian cancers (EOCs) [49]. Those workers reasonably inferred that compensatory Pol θ expression in HR-deficient tumors promoted Theta-mediated end joining (TMEJ) and likely explained the mutational scars of those cancer genomes (Fig. 1B). A recent study showed that Fanconi anemia (FA) cell lines upregulate Pol ι and rely on this Y-family DNA polymerase for viability [50] (Fig. 1C). However, the impact of Pol ι on the genome of FA cells has not yet been addressed; it will be interesting to determine whether FA cells harbor mutational signatures that are attributable to Pol ι. Indeed, it is possible that compensation for primary DNA repair defects by error-prone polymerases is a general paradigm for mutational portraits of cancer cells (Fig. 1). It will also be interesting to test whether additional tumor genomes with a primary DNA repair deficiency exhibit evidence of elevated activity of error-prone polymerases either by activating mutations or increased transcripts of a polymerase or characteristic patterns of mutation.

### (iii) Overexpression of DNA polymerase activators/pathway components

Although several studies have now described the altered expression of error-prone polymerases in cancer, only a handful of those have begun to elucidate the underlying mechanisms of those alterations. A recent study by Prodhomme et al. identified zinc finger E-box binding homeobox 1 (ZEB1, a master EMT inducing-transcription factor) as a transcriptional repressor of the *POLQ* gene [51]. Interestingly, those workers showed that ZEB1 expression restrains TMEJ pathway activity and determines the mutational spectra of different breast cancer subtypes. By analogy, other transcriptional regulators of genes encoding error-prone polymerases are also likely to have significant impact on the genome.

There are many examples of how factors determining the expression and activity of error-prone DNA polymerases are pathologically altered in cancers in ways that are likely to impact genomic stability. The TLS polymerase Pol η is pathologically degraded via at least two independent pathways in different cancers. Jung et al. showed that the E3 ligase MDM2 which is commonly overexpressed *via* amplification and other mechanisms in many tumors, targets Pol η for degradation in cultured cancer cell lines [52]. Therefore MDM2-mediated Pol η degradation provides a potential mechanism by which TLS polymerase usage might be deregulated, recapitulating the genomic instability of XP-V. In a second example, Ziv and colleagues identified an interesting mechanism by which Pol η is ablated in cancer. Those workers showed that Nucleophosmin (NPM1), a gene commonly mutated in acute myeloid leukemia (AML), interacts with the Pol η catalytic core and promotes excessive degradation of the polymerase [53]. Significantly, those workers showed that NPM1-induced Pol η degradation was associated with reduced replication fidelity of DNA templates containing Pol η cognate lesions [53].

Conversely, pathological activation of ubiquitin signaling in cancer cells has the potential to over-stimulate Pol η activity. The E3 ubiquitin ligase RAD18 mediates PCNA mono-ubiquitination and is a proximal activator of Pol η (and the other Y-family TLS polymerases). *RAD18* mRNA and RAD18 protein are aberrantly overexpressed in many cancer cell lines [54]. In cultured cells, even slight increases in RAD18 expression stimulate PCNA mono-ubiquitination, drive recruitment of Y-family TLS polymerases to replicating DNA (even in the absence of a replication obstacle or damaged DNA) [55], and promote replication across a lesion [56] or through a difficult sequence such as a fragile site [57]. Analysis of TCGA datasets reveal positive association between *RAD18* expression and overall SNV burden in several tumors including lung adenocarcinoma (LUAD), lung squamous cell carcinoma (LUSC), and kidney renal clear cell carcinoma (KIRC) [58] (Fig. 1D). In some cancer cells, RAD18 protein overexpression is due to a mis-expressed germ cell protein, the Cancer/Testes Antigen MAGE-A4 which directly binds and stabilizes RAD18 [54]. Similar to overexpressed RAD18, ectopically-expressed MAGE-A4 promotes Pol η-mediated replication of DNA templates harboring CPD [54]. Taken together it seems likely that aberrantly-expressed RAD18 contributes to TLS and mutagenesis. Different Y-family TLS polymerases have different dependencies on PCNA mono-ubiquitination for engaging the replisome. For example, the Pol η PIP box binds PCNA with high affinity when compared with the Pol κ PIP box [59]. Therefore, aberrant RAD18 activation in cancer might preferentially activate individual Y-family DNA polymerases, contributing to TLS pathway imbalance and mutagenesis.

Another E3 ubiquitin ligase, *RNF168* (the mutated gene product responsible for RIDDLE syndrome), was also identified as a potential activator of Pol η [60]. RNF168 is a key mediator of the DNA damage response which ubiquitinates chromatin in the vicinity of double-strand breaks (DSBs), thereby orchestrating the recruitment of repair proteins such as 53BP1 to sites of DNA damage [61]. RNF168 is aberrantly overexpressed in many cancers, frequently due to gene amplification [62]. Cipolla and colleagues found that overexpressed RNF168 leads to aberrant Histone H2A ubiquitination in the vicinity of DNA replication forks, and recruits excessive Pol η via direct interaction with the UBZ domain [60]. Therefore, the RNF168/H2A signaling axis represents another mechanism for pathological activation of Pol η and elevated mutagenesis in cancer cells. Taken together, it is clear that altered expression and/or activities of error-prone DNA polymerases and their proximal activators can occur in cancer cells (sometimes to compensate for other genome maintenance defects) and such changes represent a potential source of mutability. However, the gap between observing a pattern of mutagenesis in a patient sample and implicating specific polymerases in the observed mutagenesis remains to be bridged. The inherent limitations of deriving patterns of mutagenesis from in vitro experiments highlight the need for more generalizable approaches. Throughout the following sections, we enumerate and discuss experimental strategies for bridging this knowledge gap.

## Tumor mutations based on dysregulation of TLS polymerase(s)

**Fig. 2:**
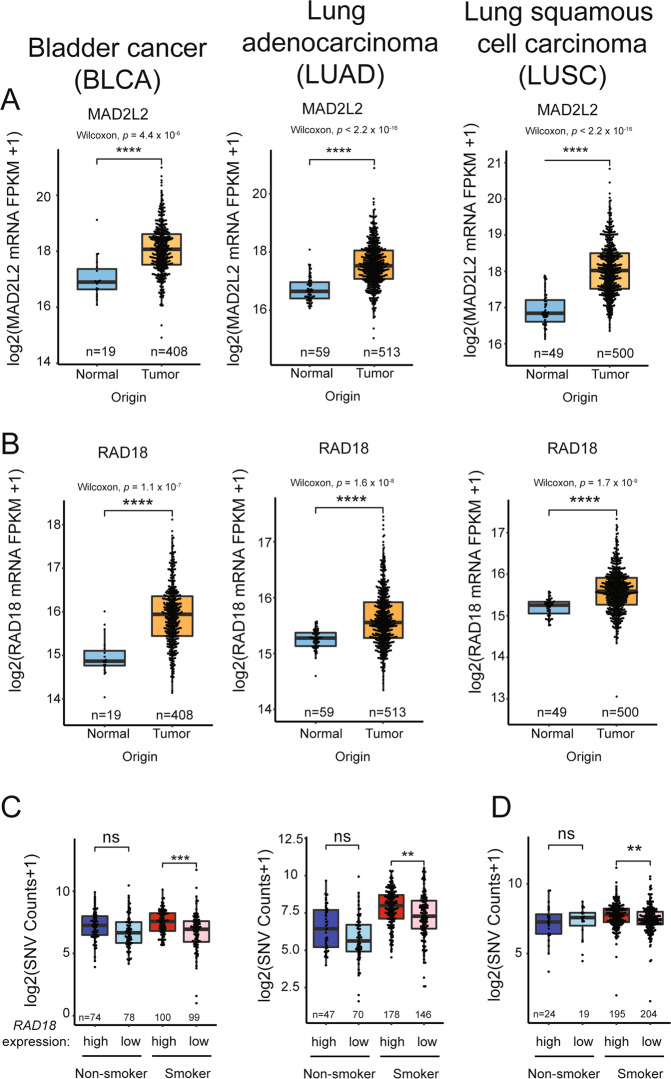
Relationship between TLS pathway genes expression and overall SNV in representative tumors. **A**, **B** Boxplots showing *MAD2L2* (**A**) and *RAD18* (**B**) expression in the indicated tumors and adjacent normal tissues. **C**, **D** Boxplots showing total SNV counts in the indicated tumor samples grouped by *RAD18* mRNA expression and smoker/nonsmoker. *RAD18* expression is indicated by “high” (upper half) or “low” (bottom half) in smokers and nonsmokers. *P* values were based on two-tailed Wilcoxon rank-sum test between groups, and were adjusted using the Benjamini–Hochberg correction for multiple tests between tumors and adjacent normal tissues, or the Holm correction for multiple tests between comparisons of gene -hi/-lo expressing samples. (*P < 0.05; **P < 0.01; ***P < 0.001; ****P < 0.0001). “*n*”, number of samples.

While there are many individual examples of how TLS DNA polymerase expression is altered in cancer cells, it is unknown whether imbalanced expression of DNA polymerases is a general source of mutations in tumors. Therefore, we have analyzed the Cancer Genome Atlas (TCGA) [63] gene expression datasets to determine whether levels of key TLS pathway genes predict or correlate with mutational load in representative cancer types. We focused on expression profiles of *RAD18* (proximal activator of all Y-family TLS polymerases), *POLH*, *POLK*, *POLI*, and *REV1* (the four Y-family TLS polymerases), and *REV3L* and *MAD2L2* which encode the two subunits of Pol ξ, the DNA polymerase involved in the extension phase of TLS. We analyzed three representative cancers for which there are large patient cohorts and mRNA expression and mutation data in TCGA, namely bladder cancer (BLCA), lung squamous cell carcinoma (LUSC), and lung adenocarcinoma (LUAD). Notably, the etiology of all three of these diseases is associated with exposure to tobacco smoke carcinogens which we reasoned might amplify patterns of mutagenesis. Table 2 summarizes the results of our analyses. Of the TLS factors profiled, only RAD18 and MAD2L2 (REV7) were overexpressed in all three tumor types (Fig. 2A, B). RAD18 overexpression correlated significantly with SNV burden regardless of smoking status in BLCA and LUAD (Fig. 2C), and also significantly correlated with SNVs in smokers with LUSC (Fig. 2D).

**Table 2:** Expression of TLS pathway genes correlates with mutational load in cancer.

	Bladder cancer (BLCA)	Lung Adenocarcinoma (LUAD)	Lung squamous cell carcinoma (LUSC)
Pol η		*Negatively* correlated with SNVs in smokers.	Downregulated in tumors *Negatively* correlated with SNVs in non-smokers.
Pol ι		Downregulated in tumors *Negatively* correlated with SNVs in smokers.	Downregulated in tumors.
Pol κ	Downregulated in tumors.	Downregulated in tumor. *Negatively* correlated with SNVs in all tumors.	Downregulated in tumors.
REV1	Downregulated in tumors.	Downregulated in tumors. *Negatively* correlated with SNVs in smokers.	
MAD2L2	Overexpressed in tumors.	Overexpressed in tumors. *Positively* correlated with SNVs in smokers.	Overexpressed in tumors.
REV3L	Downregulated in tumors.	Downregulated in tumors.	Downregulated in tumors.
RAD18	Overexpressed in tumors. *Positively* correlated with SNVs.	Overexpressed in tumors. *Positively* correlated with SNVs in all tumors irrespective of smoking history.	Overexpressed in tumors. *Positively* correlated with SNVs in smokers.

Analysis of TLS pathway genes in representative tumor types from TCGA gene expression datasets [63] as performed in ref. [58].

**Fig. 3:**
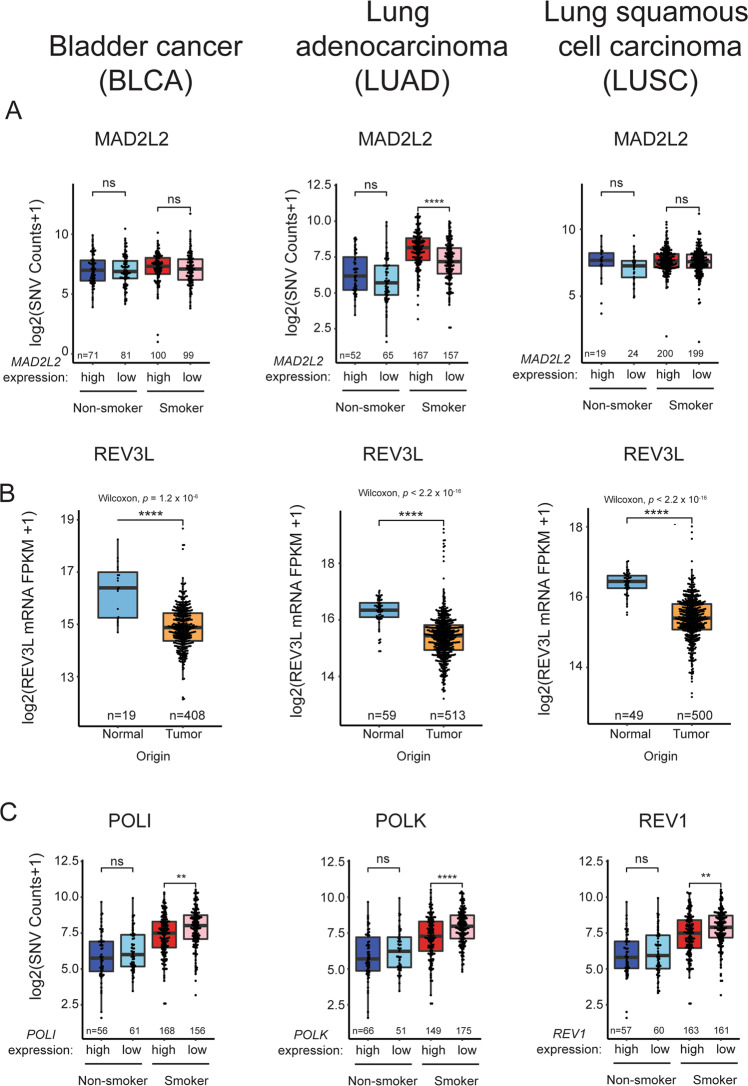
Relationship between TLS pathway gene expression and overall SNV in representative tumors. **A** Boxplots showing total SNV counts in the indicated tumor samples grouped by *MAD2L2* mRNA expression and smoker/nonsmoker. *MAD2L2* expression is indicated by “high” (upper half) or ‘low’ (bottom half) in smokers and nonsmokers. **B** Boxplot showing *REV3L* expression in the indicated tumors and adjacent normal tissues. **C** Boxplots showing total SNV counts in the indicated tumor samples grouped by *POLI, POLK,* or *REV1* mRNA expression and smoker/nonsmoker. mRNA expression is indicated by “high” (upper half) or “low” (bottom half) in smokers and nonsmokers. *P* values were based on two-tailed Wilcoxon rank-sum-test between groups, and were adjusted using the Benjamini–Hochberg correction for multiple tests between tumors and adjacent normal tissues, or the Holm correction for multiple tests between comparisons of gene -hi/-lo expressing samples. (*P < 0.05; **P < 0.01; ***P < 0.001; ****P < 0.0001). “*n*”, number of samples.

Mechanistically, it is clear that elevated RAD18 activity could promote Y-family DNA polymerase activities and induce mutagenesis. While expression of *MAD2L2* (encoding the non-catalytic subunit of Pol ξ) was elevated in all three tumors, its overexpression was only associated with increased SNVs in smokers with LUAD (Fig. 3A). In contrast with *MAD2L2*, expression of *REV3L* (encoding the catalytic subunit of Pol ξ) was significantly reduced in all three tumor types (Fig. 3B). Given that the functional Pol ξ holoenzyme requires both MAD2L2 and REV3L subunits, it seems unlikely that elevated *MAD2L2* expression alone would significantly promote TLS. Independent of its role in TLS, MAD2L2 is also a mitotic regulator which inhibits the anaphase promoting complex/cyclosome (APC/C) in prometaphase [64]. A related APC/C inhibitor, MAD2 is known to be overexpressed in tumors and contributes to mitotic defects and aneuploidy of cancer cells [65]. It is possible therefore that the overexpression of *MAD2L2* in BLCA, LUAD, and LUSC is related to tumorigenic phenotypes involving mitotic regulation but not TLS. Unexpectedly, the mRNA levels of the Y-family polymerases are generally unchanged or lower in BLCA, LUAD, and LUSC when compared with normal tissues (Table 2). In some instances, reduced expression of Y-family polymerases is associated with reduced SNV burdens as in LUAD of smokers where Pol ι, Pol κ, and REV1 levels are reduced coincident with high SNV counts (Fig. 3C).

Thus, a survey analysis of gene expression profiles in three tumor types using TCGA reveals ample evidence of imbalance in expression levels of TLS polymerases and their activators (e.g., RAD18). With the caveat that mRNA expression alone is an imperfect surrogate for protein activity, the observed changes in expression of TLS genes could significantly impact the cancer genome. For example in LUAD, the combination of high-level RAD18 with reduced Pol ι, Pol κ, and REV1 expression could lead to imbalance favoring Pol η whose error-prone replication of smoking-associated DNA damage could lead to mutations. Clearly experiments are needed to model the types of TLS pathway imbalance indicated in Table 2, and to determine whether such imbalance impacts the genome and recapitulates mutation signatures found in tumors.

**Fig. 4:**
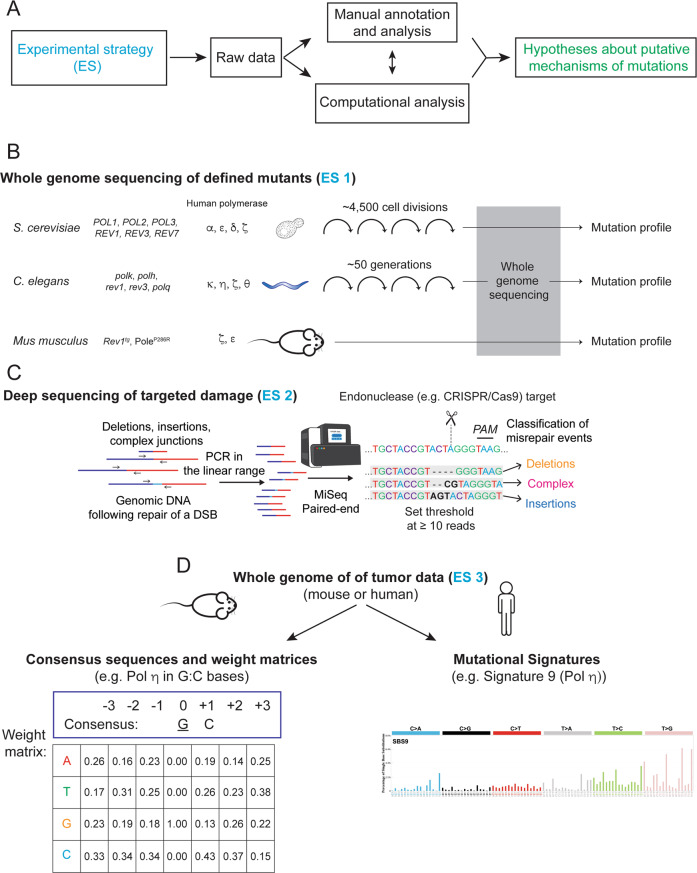
Experimental strategies for investigating polymerase-dependent mutational mechanisms. **A** Schematic description of how different experimental strategies (ES) are combined with analysis to produce mechanistic hypotheses. **B** Experimental strategy 1 (ES1) comprises whole-genome sequencing of defined mutations of model organisms including yeast [20], *C. elegans* [67, 68, 72], and mice [48, 73]. **C** ES2 illustrates a complementary approach of deep sequencing of targeted DNA damage induced by CRISPR/Cas9 in cells with different genetic backgrounds [82, 86]. **D** Consensus sequences and weight matrices [126] or mutational signatures [114] can be applied to whole-genome sequencing of human or mouse tumors [58] in ES3.

One such experimental strategy is to ablate or over-produce TLS proteins of interest in cultured cells or experimental animals, then determine the impact of those alterations on spontaneous or genotoxin-induced mutagenesis (Fig. 4). Lou et al. recently determined the contribution of Rad18-deficiency to the mutational signatures of carcinogen-induced skin cancers [58]. This study used a carcinogen (17, 12-dimethylbenz[a]anthracene or DMBA, a synthetic polycyclic aryl hydrocarbon) to induce skin tumorigenesis in experimental mice, then compared mutation patterns in clonal tumors from *Rad18*^*+/+*^ and *Rad18*^*−/−*^ genetic backgrounds. Overall, SNV burden was reduced in *Rad18*^*−/−*^ tumors when compared with *Rad18*^*+/+*^, demonstrating that *Rad18* promotes overall mutagenesis. Moreover, while COSMIC Signature 22 predominated the overall mutational portrait of *Rad18*^*+/+*^ tumor genomes, the relative contribution of this signature to the overall mutations of *Rad18*^−*/−*^ tumors was reduced by ~50% and replaced by other signatures. Therefore in a DMBA-induced carcinogenesis model, Signature 22 is Rad18-dependent. In humans, COSMIC Signature 22 is found in upper urothelial cancer (UUC) samples with known exposures to aristolochic acid, a plant alkaloid used in traditional medicines and natural remedies. Similar to DMBA, aristolochic acid induces DNA damage primarily at adenine residues and generates a mutation signature characterized primarily by AT>TA transitions. It is, therefore, possible that the aristolochic acid-associated COSMIC Signature 22 in humans is attributable to RAD18-mediated TLS. Indeed, recent data from *C. elegans* suggests that a significant proportion of aristolochic acid-induced single base substitutions can be attributed to TLS Pol η, which is recruited in a RAD18-dependent manner [66].

In the DMBA-induced carcinogenesis model, the relative contribution of COSMIC Signature 3 was higher in *Rad18*^*−/−*^ tumors when compared with *Rad18*^*+/+*^. *Rad18*^−*/−*^ tumor genomes also harbored significantly increased numbers of indels when compared with *Rad18*^*+/+*^ genomes. Taken together, the study by Lou et al. suggests that Rad18 promotes error-prone TLS across DMBA-adducted template DNA, leading to increased mutation burden and generates a specific subset of COSMIC signatures, while averting DSBs. In the absence of *RAD18*, DMBA-adducted DNA is likely processed by alternative pathways leading to different mutation signatures (e.g., Signature 3). The high levels of indels also observed in Rad18-deficient tumors might result from error-prone repair of DSBs arising via replication fork collapse, a hallmark of genotoxin treatment in TLS-deficient cells. Interestingly, in the absence of DNA damage, Rad18-deficiency in human induced pluripotent stem cells (iPSCs), did not produce a significant mutational signature [1]. The authors of this exhaustive study used CRISPR to knockout 43 DNA repair genes in iPSCs and analyze the mutational signatures produced after 15 days in culture. Though several knockouts, including the endonuclease Exo1 and E3 ubiquitin ligase RNF168, produced novel mutational signatures, in this experimental setting neither Rad18 nor the polymerases Pol θ, Pol ι, or Rev1 was among them. In a *C. elegans* study (discussed below), homologs of both Pol θ and Rev1 were found to protect the genome from endogenous sources of DNA damage [67, 68], suggesting cell-type or organismal differences in DNA repair pathway reliance or simply that an extended experimental timeframe is required to observe the activity of these polymerases.

Further experiments are necessary to identify the DNA polymerases responsible for the Rad18-dependent and -independent mutation signatures identified [40]. The contribution of specific DNA polymerases to the mutational landscape of many human tumors is not known. Starting with a tumor genome and extracting mutation patterns can be challenging because many tumor cells are notoriously genetically unstable; multiple mechanisms of mutagenesis can occur simultaneously as discussed above. An alternative approach goes in the opposite direction: starting with an experimental animal engineered with a defined genetic deficiency and observing genome-wide patterns of mutagenesis over time. Studies employing this experimental approach have provided strong evidence to link specific mutational signatures with defined mutations in polymerase genes. Results from genome-wide surveys of defined mutants are considered in the next section.

## Model organisms bearing polymerase deficiencies

Recent work in model organisms has granted insight into the role of polymerases in the accumulation of *de novo* mutations across the genome. A series of careful studies used whole-genome sequencing of *C. elegans* propagated for ~50 generations to examine the roles of TLS polymerases (Fig. 4B). The authors found that in the absence of *polk*, *polh*, *rev1*, and/or *rev3*, small deletions (50–500 base pair (bp)) orchestrated by *polq* (*C. elegans* homolog of Pol θ) with a characteristic 1 bp of microhomology and the occasional templated insertion occur randomly throughout the genome [67, 68]. Thus, compensation for TLS polymerase deficiency by *polq* leads to a characteristic signature of mutagenesis. Remarkably, the authors found naturally occurring mutations in wild-type worms that bore the signature of *polq*-mediated mutagenesis [67, 69] suggesting that TLS polymerases do not need to be genetically compromised for this mechanism to become physiologically relevant.

Subsequent studies employed a similar long-term worm propagation strategy to focus on endogenous mutagenesis near difficult to replicate DNA such as G quadruplexes (G4). In the context of G4 DNA, *polq* was again implicated in a class of deletions narrowly restricted to 50–300 bp, with a single bp of microhomology and occasionally containing templated insertions [70]. Notably, the boundaries of these *polq*-dependent deletions occurred overwhelmingly at the edge of G4 DNA structures [70, 71].

Establishing the spontaneous mutagenic profile of polymerases allows comparison with outcomes of treatment with genotoxins that induce DNA lesions. In the presence of genotoxins that alkylate or crosslink DNA, *polq* was also found to play an essential role in mutagen-induced deletions in *C. elegans* [72]. As determined by analysis of ~7000 *polq*-dependent deletions, these genomic scars were predominantly 50–1000 bp in size, contained 1 bp of microhomology and roughly one quarter had small insertions of ~5 bp, a mutagenesis phenotype reminiscent of *polq*-dependent endogenous mutagenesis. A recent study iterated this approach of long-term propagation of *C. elegans* to comprehensively profile the interactions between error-prone polymerase activity and many different types of DNA damage. In this tour de force study, the authors investigated the interactions between 11 different genotoxins and 54 genotypes representing 8 different DNA repair pathways that included 6 error-prone polymerases [66]. These results corroborated many earlier findings and clearly demonstrated the complex interactions of variables in mutagenic processes. For example, *polh* and *rev3* were found to induce base substitutions at alkylated bases, while *polk* prevented this mutagenesis [66]. These observations have implications for defining therapeutic strategies using alkylating agents in tumors with Pol η or Pol κ dysregulation (Table 2).

While *C. elegans* lacking rev1 can be propagated for 50 generations to obtain a mutational profile [68], REV1-deficiency is challenging to study in mice because *Rev1*^*−/*−^ mice exhibit growth delays and compromised fertility. However, a recently described REV1 transgenic mouse provides evidence to support a role for REV1 in genome-wide mutagenesis and tumorigenesis. Overexpression of REV1 accelerates the development of intestinal adenomas in a carcinogen-induced tumor model. Further, it increases the point mutation frequency in exon 3 of the *Ctnnb1* gene within the murine tumors [48]. This finding recapitulates some aspects of the phenotype in *C. elegans* in which *rev1* promotes spontaneous point mutations but also suppresses small deletions [68]. Whole-genome sequencing (WGS) of tumors in REV1^tg^ mice will be informative as a comparison to human tumors exhibiting REV1 upregulation (e.g., gliomas [45]). Interestingly, our analysis (described in the previous section) finds that REV1 expression levels are negatively correlated with SNV burden in human lung adenocarcinomas from smokers (Table 2), suggesting that the role of REV1 in tumor mutagenesis may be context dependent.

In an elegant translation of clinical observations to a faithful laboratory model, mice engineered to express a human tumor-derived allele of Pol ε (P286R) led to spontaneous malignancies of diverse lineages [73]. WGS of primary tumors revealed a high frequency of point mutations (10–100/Mb) that was comparable to carcinogen associated human tumors such as lung. *Pole*^*P286R*^-driven murine tumors exhibited a high incidence of C>A and C>T substitutions with a bias for TCT flanking base context, consistent with COSMIC Signature 10 [74] described in human cancers with germline or spontaneous mutation of Pol ε. The mouse model of Pole-driven tumorigenesis provides a remarkable molecular recapitulation of the corresponding human malignancies and therefore is an ideal experimental model to test the mutagenic contribution of additional environmental or genetic factors such as chemotherapy and dysregulated repair pathways [73]. Indeed, a recent study examining *Pole*^P286R^-dependent endometrial carcinomas found that mismatch repair (MMR) deficiency cooperates with *Pole*^P286R^ to accelerate tumor progression and increases indel mutations while shifting the spectra of point mutations from mostly T>G in *Pole*^P286R^ to mostly C>A [75]. The mutagenic patterns described in long-term propagation of genetically defined *C. elegans* strains and a growing number of mouse models have already begun to enable the recognition of similar mutation patterns in cancer genomes and the mechanistic explanations for these patterns.

## Deep sequencing of targeted DNA damage

An alternative approach to the challenge of identifying global patterns of mutagenesis is to perform an in-depth profile of the specific genomic region surrounding targeted DNA damage (Fig. 4C). Advantages of this experimental strategy include tractability, acceleration of experimental timelines, and the ability to query different genetic dependencies and contexts with relative ease. In particular, deep sequencing of repaired loci simplifies the problem in two ways: (1) it limits analysis to a small genomic region and (2) focuses on mechanisms of mutagenesis that are downstream of damage induction. Coupling next-generation sequencing (NGS) to CRISPR/Cas9 technology has rendered this approach feasible in a broad range of contexts and more easily accessible with the advent of user-friendly interfaces [76, 77]. In mammalian cells, DSBs are primarily repaired by three pathways: homologous recombination (HR), classical non-homologous end joining (c-NHEJ) and a pathway variously termed microhomology mediated end joining (MMEJ) or alternative non-homologous end joining (alt-NHEJ) that relies upon the activity of Pol θ. As targeted DNA DSBs are predominantly repaired either by c-NHEJ or alt-NHEJ [78–80], current applications of amplicon sequencing interrogate the mechanistic underpinnings of these pathways including the roles of polymerases therein (Table 1).

An early iteration of this experimental strategy demonstrated its promise using zinc finger nucleases and Sanger sequencing to show that human cells deficient in the c-NHEJ factors LIG4 and/or XRCC4 repair DNA DSBs with reduced indel frequency and increased reliance on microhomology [81]. Applying NGS to amplicons subsequently led to a comprehensive description of Pol θ’s role in repair of DSBs [82]. Pol θ-deficiency in a wild-type background modestly reduced deletion events that rely upon short microhomologies [82, 83]. However, the tractability of this approach permitted evaluation of Pol θ activity in additional repair-deficient backgrounds and revealed that Pol θ is responsible for a larger proportion of repair when NHEJ is compromised. In this context, Pol θ-dependent repair is characterized by deletions of intermediate size (5–50 bp) and microhomology (MH) usage [82]. Further, Pol θ-dependent microhomology usage was limited to within 15 bp of the DSB [84]. A subsequent study using human cancer cells with disrupted Pol θ, confirmed that Pol θ-dependent repair exhibits an increased reliance on MH and that templated insertions with MH are enriched in Pol θ-dependent repair of distal DSBs [19]. When such large numbers of repair events are recovered, analysis of rare events is possible [85]. For example, Pol θ was found to mediate insertions both from proximal sequences and less commonly but robustly, from across the genome [82]. Notably, the profile of Pol θ-dependent mutagenic repair of targeted DSBs is similar to the *polq*-dependent mutagenesis phenotype observed in *C. elegans* [70]. The Pol θ-dependent signature of mutagenesis revealed by applying NGS to amplicons contributed to the identification of the role of Pol θ in HRD tumor mutagenesis [19].

Amplicon NGS has also been used to assess the role of the replicative Pol δ in repair of DSBs [86]. Similar to Pol θ, Pol δ depletion in a wild-type background had a modest phenotype: overall, imprecise repair in Pol δ-depleted cells exhibited fewer deletions and more insertions with a decreased reliance on microhomology. The DSB repair phenotype suggests that Pol δ plays a role in an error-prone alt-NHEJ mechanism; this was bolstered by the observation that far fewer rearrangements in Pol δ-depleted cells exhibited the end processing characteristic of alt-NHEJ [86]. Again, the experimental tractability enabled evaluation of the genetic interaction between Pol δ and LIG3, a key factor in alt-NHEJ. The repair phenotypes of single and combined deficiency in Pol δ and LIG3 indicate that the two factors act in distinct alt-NHEJ mechanisms. The flexibility of the NGS amplicon strategy allowed complementation with separation of function mutants of Pol δ. This study found evidence to support roles for both the DNA synthesis and exonuclease activities of Pol δ in error-prone repair of DSBs.

A distinct advantage of amplicon NGS is that it can analyze repair of artificially introduced extrachromosomal plasmids or DNA fragments, permitting complete control over the DNA sequences proximal to the break. A *Drosophila* study focused on the contribution of immediate sequence around the DSB tested the role of secondary DNA structures including loops and hairpins by using the endonuclease I-*Sce*I to cut a plasmid with carefully designed flanking sequences [87]. Remarkably, a single nucleotide change abrogated hairpin formation and was sufficient to dramatically alter the spectra of repair outcomes. Notably, nucleotide changes up to 30 bp distal to the DSB were found to influence repair outcomes indicating that secondary structure plays a significant if incompletely understood role in NHEJ-mediated repair of DSBs. Similarly, to achieve systematic variation of the break site sequences, Carvajal-Garcia et al. introduced DNA fragments instead of using CRISPR/Cas9. Careful design of these DNA fragments with microhomologies at varying distance from the break revealed that Pol θ scans 15 bp bidirectionally from the broken ends to find microhomology and that AT-rich sequences are more prone to templated insertion [84], likely because of their reduced thermodynamic stability with complementary sequences. At endogenous sequences, careful design of CRISPR/Cas9 targets can also afford significant control over the DSB proximal sequences as in a recent study of Pol α primase. By cleverly targeting DSBs to Pol α “deserts” (i.e., sequences that prevent Pol α primer initiation), the authors found evidence that Pol α is responsible for tandem duplications at DNA break termini [88].

Introduction of pre-cut extra-chromosomal fragments as in [84] is one way to address an inherent limitation of this technique: the inability to distinguish between uncut loci and perfect repairs. Whether perfect repair of Cas9-mediated DSBs is a significant outcome is unclear with a recent study reporting that it is relatively minor at many loci [79]. However, extra-chromosomal DNA fragments do not fully approximate broken chromosomes because they likely lack most chromatin proteins. Therefore, an orthogonal strategy to assess minimally processed repairs (i.e., “perfect repair”) using amplicon NGS is to examine the sequence of chromosomal rearrangements [81, 83, 86, 89] with the caveat that the long- and short-range joining of DSBs may have slightly different genetic dependencies.

Evidence is accumulating that DNA secondary structures can influence mutagenic repair outcomes [84, 87]. However, in experiments relying upon targeted endonucleases, this influence may occur by interaction with endogenous repair factors, exogenous genome editing machinery or both. Indeed, CRISPR/Cas9 target sequence was among the strongest predictors of repair outcomes following DSBs across different genomic contexts or cell lines [78] although some modest cell-type specific differences were observed such as a permissiveness for larger insertions in stem cells [80]. These studies support the notion that the experimental framework of amplicon NGS is best applied to identical target sequences in different genetic or genomic contexts. However, by comparing the frequency of the commonest indel between diverse gRNAs one study suggests a possible framework for comparing repair profiles across different gRNA sequences [90]. The recent advent of tools that perform in silico prediction of the repair outcomes for a given gRNA target sequence [80, 91] will be very useful to guide selection of targets to investigate the contribution of either c-NHEJ or alt-NHEJ.

To leverage NGS of amplicons for a broad test of chromatin contexts on DSB repair pathway usage, a recent study cleverly used multiplexed integrated reporter sequences at >1000 random sites genome-wide [92]. This approach distributed the same CRISPR/Cas9 target site throughout the genome to avoid differences in repair outcomes that result from different target sequences. Specific indels resulting from imprecise repair of the reporter were characteristic of either NHEJ pathway activity or Pol θ-dependent alt-NHEJ [79, 92]. Deep sequencing of these genomic loci after repair revealed that NHEJ activity increased in euchromatic regions and Pol θ-dependent alt-NHEJ predominated in heterochromatic regions. The remarkable profile generated using this technique enabled complex kinetic investigations of context: NHEJ predominated at early timepoints with a later shift towards alt-NHEJ that was more dramatic in heterochromatin [92] indicating that local chromatin environment could play an integral role in the recruitment of Pol θ.

While most of these studies have leveraged NGS of amplicons to study mutagenesis at DSBs, a recent study examined single-strand breaks (SSBs) instead and found that several polymerases contribute to mutagenesis at these lesions [93]. Depletion of BRCA2 unexpectedly led to dramatically increased frequency of insertions at an SSB, permitting the authors to test polymerases for their role(s) in this specific type of mutagenesis. They observed that the Y family polymerase REV1 orchestrates insertion of a G nucleotide opposite a nick in the absence of BRCA2 [93]. In contrast, REV3, a component of Pol ξ, suppressed these insertions. At SSBs, Pol θ suppressed 1 bp insertions while promoting longer insertions. The frequency of single nucleotide variants (SNVs) in proximity to SSBs also increased dramatically in the absence of BRCA2 with REV1, REV3, and REV7 all reported to play modest roles in this phenomenon. Surprisingly, amplicon sequencing identified Pol θ as the major player in generating SNVs near SSBs and DSBs [93]. Taken together, these amplicon sequencing results reveal the significant mutagenic potential of SSBs which has long been questioned. These innovative applications of amplicon sequencing highlight that there remains much to be discovered with these experimental methods.

Despite the experimental power and robustness of deep sequencing genomic scars of targeted DNA damage, several challenges remain including artifacts that may be introduced by the endonuclease. It is imperative to consider the ways in which repair of endonuclease-induced DNA damage may not represent repair of naturally occurring lesions. The frequently observed single bp insertions likely result from Cas9 remaining bound on the PAM-proximal side while a staggered break is filled in with a single bp insertion that repeats the PAM-distal nucleotide [80, 89–91, 94]. In budding yeast, this insertion was dependent upon *Pol4*, a homolog of mammalian Polymerase β (Pol β) [94]. Despite that this artifact may result from Cas9-generated overhangs, Pol β is reported to play a role in DSB repair in mammalian cells [86, 95]. This Cas9-specific artifact depends heavily on sequence context [83, 90] and is particularly dominant in the repair profile when the surrounding sequences do not contain microhomologies [91].

In addition, repair outcomes were significantly different for the same DSB with Cas9 in opposite orientations, indicating that CRISPR/Cas9 itself may interact with endogenous DNA repair factors [94]. Moreover, choice of genome editing machinery can influence repair outcomes even when queried at the same DSB [83, 96, 97]. Indeed, both the histone chaperone FACT [98] and RNA Polymerase II [99] can remove Cas9 from DNA suggesting that collision with DNA polymerases might dislodge Cas9 as well. While at the break, Cas9 may hide the lesion from the cellular repair machinery thereby delaying activation of repair pathways or asymmetrical Cas9 binding may lead to directional bias in repair [97, 100]. One additional limitation of many NGS amplicon studies is the relatively small size of PCR amplicons which in turn limits the size of the sequence modifications that can be observed. One strategy to expand the window is to use PacBio sequencing technology to capture larger events [101, 102] that may occur at non-negligible frequencies in human cells [102].

A recent study placed Cas9 target sequences in two categories: “precise” at which a single indel predominates the repair and “imprecise” at which numerous less frequent repair events occur [90]. Cas9 gRNA’s with high efficiency of cleavage tend to also exhibit a characteristic indel that accounts for the majority of the repair outcomes while gRNA’s with lower efficiency exhibit a greater diversity of repair outcomes [90] suggesting that less efficient gRNAs may be better suited to measuring repair outcomes downstream of a CRISPR/Cas9 DSB.

As noted above, the tractability of amplicon NGS to measure DSB repair outcomes lends itself to additional experimental conditions. To date, DSBs have been the main focus of amplicon NGS but the patterns of mutagenesis caused by DNA single strand breaks [103] have only begun to be examined. Similarly, coupling amplicon NGS with protein adducts that stall replication forks and lead to fork collapse [104] may elicit revealing patterns of mutagenesis. In addition, use of Cas9 fused to a light-activated photosensitizer that generates reactive oxygen species in proximity to DNA could enable mutagenic analysis of targeted ROS damage [105, 106] that might more faithfully recapitulate some forms of endogenous damage. Another possibility is to fuse Cas9 to a downstream repair effector in order to force the first step of repair to engage a particular pathway. This paradigm has been demonstrated in Cas9-TREX2 fusions and the resulting bias towards larger deletions [80], Cas9 fusions to CtIP [107] and additional repair factors [108] to query mechanistic steps downstream of an early pathway commitment step. In addition, results from NGS experiments can be used to guide design of repair pathway specific assays to quantify the contribution of multiple competing pathways [109]. Much remains to be studied with the tractable approach. While NGS amplicons focus on a comparatively small genomic window of ~250 bp, mutable motifs and mutational signatures, discussed in the next section, focus on an even smaller genomic window but derive their power from considering statistical support for association of these short motifs with mutations throughout the genome.

## Approaches to identify mutagenic context features

Mutation frequencies vary significantly along nucleotide sequences such that mutations often concentrate at certain positions called hotspots [110]. Mutation hotspots in nucleotide sequences reflect intrinsic properties of the mutation process. For example, sequence context specificity manifests itself at the level of interaction between mutagens, DNA/RNA, and the action of the repair and replication machineries. Analyzing the local nucleotide sequence context of mutations can reveal information about the molecular mechanisms of mutagenesis [111].

Many studies have identified specific DNA sequence patterns associated with elevated mutation frequency. For example, repetitive sequences such as homonucleotide runs, direct and inverted repeats, and microsatellite repeats are involved in specific types of high frequency mutational events (reviewed in ref. [111]). For these mutation hotspots, the exact DNA sequence is less critical than the fact that a sequence motif is repeated. Alternatively, mutation hotspots emerge due to the influence of neighboring nucleotides [112]; those neighboring nucleotides are described by mutable motifs [111]. The simplest example of a mutable motif is the CpG dinucleotide in human genes, mutations of which likely result from deamination of methylated cytosines [113]. Mutable motifs constitute a powerful approach to study mutagenesis because, in many cases, they directly represent fingerprints of interactions between nucleotide sequences and repair, replication, or modifying enzymes, thereby providing clues as to the underlying molecular mechanisms of mutation [114].

The consensus sequence is the calculated sequence of most frequent residues found at each position in a sequence alignment and the most straightforward implementation of a mutable motif. One example of a mutable motif derived with this approach is YCG/CGR (Y = T/C, R = A/G, mutable positions are underlined) which is found to be hypermutated in human normal and cancer skin cells [115] presumably as a result of exposure to UV radiation. Similarly, applying the consensus approach to mutation spectra resulting from in vitro Pol β error-prone DNA synthesis reveals the mutable motif GTT (T>G mutations) [110]. The characteristics of the mutations at GTT sites suggest that certain base substitution and deletion errors result from dislocation of template bases rather than from direct mispair formation by Pol β [110]. The examples discussed above demonstrate the utility of manual curation of mutations in human genes (Fig. 4A).

**Fig. 5:**
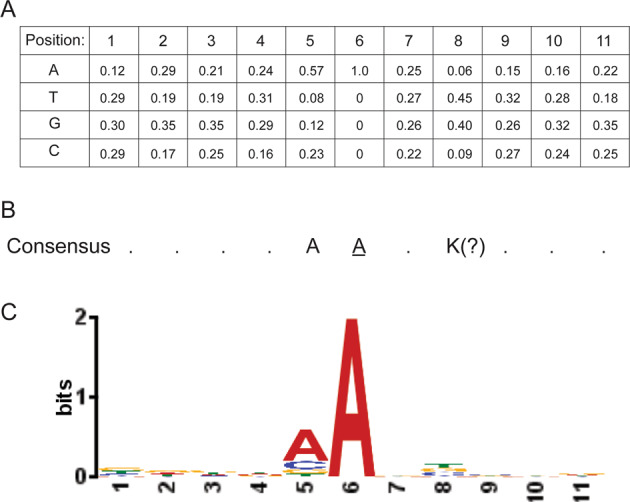
Mutable motif of the DNA context of mutations induced by Pol θ in A:T sites. **A** Frequencies of nucleotides in positions 1–11; position 6 is the site of the mutations. **B** Consensus sequence (frequencies of nucleotides were used as input and position 8 is an ambiguous position, K = T or G). **C** Shannon information content represented by the logo description of mutable motifs was constructed using the MEME website (https://meme-suite.org/meme/tools/meme).

However, in most cases consensus sequences of mutational context are computationally derived from sets of aligned mutated sequences represented by position-specific frequency matrices [114]. An example of the Pol θ frequency matrix and mutable motif is shown in Fig. 5A–C. Another example is the mutable motif of Pol η, WA / TW (W = A/T), which is derived from alignment of highly mutated sequences [116]. Pol η mutable motifs are well-studied because this polymerase performs translesion DNA synthesis and functions as the A/T mutator in vertebrate immunoglobulin genes (Ig) [116, 117]. To investigate the role of Pol η in cancer mutagenesis, human somatic mutations derived from normal and cancer cells using data from the International Cancer Genome Consortium (ICGC, [118]) and TCGA [63] were examined for the mutable motif characteristic of Pol η [119]. A significant excess of single and tandem somatic mutations within known Pol η mutable motifs was noted in skin cutaneous melanoma as well as in many other types of human cancer including some leukemias and lung and ovarian tumors [119], suggesting that Pol η-dependent somatic mutations in A:T bases are common features of tumorigenesis even in the absence of UV-mutagenesis.

Mutable motif analysis has been leveraged to address discordant data on the role of Pol θ in somatic hypermutation (SHM). While early studies in Pol θ-deficient mouse strains implicated Pol θ in SHM of Ig genes [120, 121], this putative role of Pol θ remained controversial [122]. Notably, mutable motif analysis of somatic mutations in cell lines derived from germinal center B cells supported a role for Pol θ in SHM of Ig genes [123, 124]. Further, these studies identified the likely consensus sequences of Pol θ mutable motifs as ADK / MHT (D = A/G/T; K = G/T; M = A/C; H = A/C/T) and AA/TT [123, 124]. Taken together, a putative function of Pol θ as an additional A/T mutator in Ig genes is supported by mutable motif analysis and requires further investigation.

The application of weight matrix is the further development of the consensus “motif” approach. A mononucleotide weight matrix is a simple and straightforward way to present the structure of a functional signal, such as a short nucleotide sequence, and to calculate weights for the signal sequence [125]. The weight matrix technique is different from position-specific frequency matrices (Fig. 5) discussed above because it is based on normalizations of nucleotide frequencies. Each weight matrix is a visual representation of a mutagenic motif that includes information on the normalized frequency, or weight, of the A, T, G, and C bases in each of the 6 positions surrounding the detected sites of mutation (3 bases downstream and 3 bases upstream) (Fig. 4). One of the simplest weight matrix normalizations takes into account the nucleotide composition of the target sequences in which mutations have been detected [121]. Rogozin et al. [122, 123] used the mean nucleotide frequencies of positions −5, −4, +4, and +5 for this normalization procedure. While other normalization approaches are possible for position-specific frequency matrices (Fig. 5), the strategy described above is the most frequently used [121]. This technique was recently applied to the nucleotide context of somatic mutations in multiple tumor types to investigate the role of AID and APOBECs, members of a family of DNA and RNA editing cytosine deaminases [126]. Six different AID/APOBEC mutable motifs were derived with each matrix containing information on the normalized frequency of the A, T, G, and C bases in each of the 6 positions surrounding the detected mutation (3 bases downstream and 3 bases upstream. This analysis confirmed that while mutational footprints of APOBECs-1, -3A, -3B, and -3G are prominent in many cancers, mutable motifs characteristic of AID, the primary SHM enzyme, are the most widespread feature of deaminase-dependent somatic mutation spectra in cancer genomes [126].

A subsequent study applied the weight matrix approach to mutable motifs associated with Pols θ and η [127]. An example of a matrix for Pol η (mutations in G:C bases) is shown in Fig. 4D. Further, analysis of genome-wide methylation profiles and somatic mutations in B-cell derived lymphomas using weight matrices suggested the functional importance of interplay between mutagenesis induced by AID, Pols θ and η in cancer and (de)methylation processes [127]. Control experiments that allowed estimates of predicted error rates lent even further support to the contention that the weight matrix technique is a reliable method to delineate and study mutable motifs in cases when some positions of mutable motifs cannot be easily described by the consensus approach [126, 127]. An example of such ambiguity is shown in Fig. 5 for Pol θ. It is not clear whether position 8 (which shows a sign of conservation, Fig. 5C) should be included in the consensus sequence (Fig. 5B). In many cases it is difficult to confidently delineate mutable motifs using the consensus approach owing to the lack of objective inclusion criteria for position-specific context features to mutable motifs [127]. Thus, the weight matrix approach, which utilizes information contained in all studied positions, is likely to be a more straightforward way to describe mutable motifs than the consensus approach [127].

Analysis of mutable motifs is directly associated with molecular mechanisms of mutations as in the well-studied enzymatic activities of error-prone DNA polymerases. However, a shortcoming of this approach is that a limited number of experimental datasets describing repair, mutation, or replication processes and enzymes in vivo is available. In many cases, this data is derived from in vitro experiments that cannot capture all DNA context features of the studied polymerases such that results must be interpreted with care. However, if derived mutable motifs accurately represent in vivo DNA context specificity of the studied repair, mutation, or replication enzymes, they produce accurate estimates of the overall impact of specific mutagenic enzymes in large scale studies of somatic mutations in various cancers [128]. One successful example of a consistent mutable motif derived from in vitro and in vivo experiments is Pol η context specificity [101, 102] discussed above.

Another fruitful direction in cancer research is the simultaneous derivation of multiple informative mutational signatures from analysis of a single tumor type. This approach is complementary to analyses of mutable motifs and called the mutational signature technique [129, 130]. As it is usually not possible to define the DNA strand on which a mutation occurred (e.g., distinguishing C>T mutations from G>A mutations on the opposite strand), there are six types of substitutions for analysis. Therefore, considering two nucleotides in the positions flanking the mutation, there are 96 context-dependent possibilities [131]. For multiple patients and/or samples, their context dependent mutations can be represented in the form of a nonnegative matrix X, where columns correspond to samples and rows represent context-dependent mutation types [131]. The mutational signature techniques solve the problem of finding two matrices, W and H, as a result of decomposition of X ~ WH, where W corresponds to mutational signatures, and H corresponds to exposure of samples to mutational processes described by mutational signatures [130, 132].

An example of a mutational signature (Mutational Signature 9) associated with Pol η is shown in Fig. 4D [132]. Signature 9 has been found in chronic lymphocytic leukemia and malignant B-cell lymphomas [132]. Interestingly, Signature 9 has a higher frequency of T:A>G:C transversions as compared with T:A>C:G (Fig. 4D). However, an excess of T:A>G:C transversion was not previously observed in Pol η mutation spectra [116]. This discrepancy suggests that Signature 9 may reflect context features of two or more mutational mechanisms that were merged together during classification. The possibility of merging two mutational mechanisms in a single signature illustrates an inherent challenge of the mutational signature heuristic annotation by means of manual analyses. Moreover, as discussed above, manual analyses can be helpful in understanding the mechanisms of mutations (Fig. 4A**)**. Additional criteria can also help to delineate molecular mechanisms of mutations. Supek and Lehner [133] demonstrated that clustered mutations that likely arose from the same mutagenic event provide a more precise fingerprint of mutagenic processes. For example, clustered mutations of A>G in the WA context (W = A/T) defined a mutational signature consistent with Pol η activity [133]. Notably, this clustered Pol η mutational signature was correlated with H3K36me3, a histone modification associated with active chromatin and contributed substantially to the mutational load in lymphoid tumors [133].

Given the complementary strengths of mutable motifs and mutational signatures (discussed above), merging these two approaches is likely to be a reliable strategy to study molecular mechanisms of mutations (Fig. 4**)**. An attempt to implement this strategy was described by Temiz et al. [134]. The authors presented a 32 × 12 mutation matrix capturing the nucleotide pattern two nucleotides upstream and downstream of the mutation [134]. A somatic autosomal mutation matrix (SAMM) representing tumor-specific somatic mutations and four mechanistic template mutation matrices (MTMMs) representing estimated mutation patterns for (1) oxidative DNA damage, (2) UV-induced DNA damage, (3) (5 m) CpG deamination, and (4) APOBEC-mediated cytosine mutation was constructed. MTMMs were mapped to the individual tumor SAMMs to determine the contribution of each mutational mechanisms [134]. In this analysis of 909 tumors, 92% of the SAMMs were correctly assigned to one of 11 tissues of origin, while only ~8% had an undetermined tissue of origin [134]. Thus, although tumors from different tissues may share mutation patterns, their SAMMs often display signatures that are characteristic of specific tissues. This work marks the first attempt to merge mutational signatures and mutable motifs into an integrated system to study mechanisms of mutations in cancer cells.

In the future, analyses of mutable motifs and mutational signatures are likely to play a growing role in studies of somatic mutations in cancer. These approaches could be even more informative when they are combined with additional experimental frameworks such as patient-derived xenografts and clinical data integration [135, 136]. Application of mutable motifs and mutational signatures can help to delineate cancer driver genes and even be used to identify cancer biomarkers and drug targets [127, 135–137].

## Future directions

There have been tremendous recent advances in our understanding of mutagenic DNA polymerases and analyzing mutational patterns in genomes. However, the underlying mechanisms that give rise to most mutational signatures in cancers have not definitively been described [74]. Remarkably, almost one-third of human tumors exhibit mutational patterns of unknown etiology [2]. It is clear that mutagenesis is dictated by the interaction of specific DNA lesions or endogenous obstacles with error-prone DNA polymerases and repair mechanisms. Efforts have begun to examine all of these interactions [66] and ultimately, to explain all mutational signatures it will be necessary to model many more interactions.

Extracting mutational signatures from a specific subset of mutations (e.g., clustered) has led to the identification of a signature that is more closely aligned with the in vivo data than signatures generated without regard to mutation proximity [133]. Many TLS polymerases play key roles in replication of endogenous DNA obstacles such as fragile sites and structured DNA [138] that are defined by their DNA sequence. Therefore, efforts to identify mutational signatures that occur specifically within these DNA elements may be productive.

While curating mutational signatures based solely on sequence context has been enormously informative and has propelled the field forward, this approach is likely to give an incomplete picture of susceptibility to mutagenesis. Additional factors including the local chromatin environment, the presence of secondary structures, a larger window of DNA sequence context, and chromosomal position within the nucleus are likely relevant to mutagenesis as well. These factors could impact mutagenesis by influencing both susceptibility of DNA to damage [139, 140] and access by repair factors [141–144]. Consideration of the above variables is needed in future studies to address a larger number of mutational signatures. The complementary experimental strategies described in this review coupled with future innovations will enable the field to tackle the ambitious goal of unraveling mechanisms of mutagenesis in all human tumors.

## Methods

Lung adenocarcinoma (LUAD), bladder urothelial carcinoma (BLCA), and lung squamous cell carcinoma (LUSC) were selected for evaluation of relationships between TLS gene status and genome instability in human tumors. TCGA datasets containing RNA expression, mutation, genomic alteration (downloaded on 20 March 2019), and clinical information (downloaded in May 2019) for these tumors were from the TCGA data portal (https://portal.gdc.cancer.gov). Specific datasets used in this study include: (i) HTSeq-FPKM-UQ gene expression quantification (for those duplicated samples from the same patient, the sample with higher gene expression was chosen for downstream analysis), (ii) somatic mutation aggregated and masked by Mutect2 and organized as MAF files publically available and (iii) smoking information for most subjects in LUAD, LUSC and BLCA patients. To avoid zeros when using log scale for display, log2(FPKM + 1) was used to display gene expression data obtained by RNAseq.

### Statistical analysis

R (version 4.0.3) was employed for data analysis and presentation. Groups were compared with two-tailed unpaired two-sample Wilcoxon rank-sum test using *wilcox.test*, in that alternative = “two.sided” and paired = FALSE. *P* values of the comparisons between tumor and adjacent normal samples were adjusted using the Benjamini–Hochberg correction for multiple tests among the 7 genes related to TLS pathway (POLH, POLI, POLK, REV1, MAD2L2, REV3L, and RAD18) by function *p.adjust* in *stats* package, in that method = “BH”. *P* values of the comparisons of SNVs between high/low expression level of TLS genes within smokers/non-smokers were adjusted for the same 7 genes in TLS pathway using Holm method by function *p.adjust*, in that method = “holm”.
